# Developing design principles to standardize e-commerce ecosystems: A systematic literature review and multi-case study of boundary resources

**DOI:** 10.1007/s12525-022-00558-8

**Published:** 2022-07-09

**Authors:** Tobias Wulfert, Robert Woroch, Gero Strobel, Sarah Seufert, Frederik Möller

**Affiliations:** 1 University of Duisburg-Essen, Universitätsstraße 2, 45141 Essen, Germany; 2 TU Dortmund University, Joseph-von-Fraunhofer Str. 2-4, 44227 Dortmund, Germany; 3 Fraunhofer ISST, Emil-Figge-Straße 91, 44227 Dortmund, Germany

**Keywords:** Platform Ecosystem, E-Commerce, Boundary Resources, Standardization, Design Principles, Multi-Case Study, A100

## Abstract

Platform ecosystems have captured a variety of markets, enabling coordination, transactions, and value co-creation between independent actors. A focal platform constitutes the central nexus of e-commerce ecosystems and fosters the interaction among ecosystem participants through their boundary resources. Standardizing these interfaces simplifies ecosystem entry for developers and increases the number of participants propelling the network effects, and thus the overall value of the ecosystem. Currently, there is a lack of prescriptive design knowledge guiding platform owners in designing successful e-commerce ecosystems. Addressing this issue, we followed a dual approach, reporting on a systematic literature review in which we identified design requirements and complemented these with a multiple-case study on selected e-commerce ecosystems. Aggregating the requirements resulted in six meta-requirements and 19 design principles that foster the standardization of focal e-commerce platforms. Our design principles simplify the development of complements and enable multi-homing for developers due to possible standardization across ecosystems.

## Introduction

Platform ecosystems have already captured markets such as accommodation, transportation, music, and retail, with popular focal platforms such as Airbnb, Uber, Spotify, and Amazon (Choudary, 2015; Evans & Schmalensee, 2016; Parker et al., 2016). As the largest platform in electronic commerce (e-commerce), Amazon generated US$340 billion in revenue from product and service sales in the fiscal year 2020, with more than 60% of revenues resulting from commission fees from third-party sellers on its marketplace (Amazon, 2019, 2021). Across industries, the revenue realized by ecosystem participants is up to three times higher than the revenue generated by the focal platform (Delteil et al., 2020). Business ecosystems facilitate the interconnectivity of participants within and across previously independent markets. These autonomous participants maintain their relationships with each other using information technology in e-commerce ecosystems. They emerge around focal platforms employing various business models (e.g., marketplaces, auctions) with additional types of participants, such as manufacturers, retailers, and content providers. Choosing a platform strategy is currently a common approach to commercialize digital products and has been adopted by numerous companies (Foerderer et al., 2019). The digital channel simplifies the interactions among ecosystem participants and reduces transaction costs (Nicola et al., 2020). Therefore, the success of e-commerce ecosystems depends on exploiting network effects and their strength strongly correlates with the size of the ecosystem (Briscoe et al., 2006). Thus, a platform provider must increase the number of actors from different markets by attracting external developers as participants to foster generativity and maximize ecosystem growth (Parker et al., 2017).

Establishing consensus among independent ecosystem participants, standards amplify e-commerce ecosystems by ensuring a proper value proposition (Adner, 2017; Papachristos & van de Kaa, 2021; Staudenmayer et al., 2005). Technical standards are defined as “the characteristics of a product as well as its level of performance, safety, or quality” (Viardot et al., 2021, p. 11). Standards can be defined generally and within e-commerce ecosystems by standard-setting organizations or competitive market forces that most likely are the platform owners (de facto standard) or enforced by law (de jure standard). Within e-commerce ecosystems, standardization is applied to achieve a “winner-takes-all” strategy, often involving quasi-monopolies (e.g., Amazon) that can only be broken by law (Shapiro & Varian, 1999; Viardot et al., 2021). In recent years, many standards have been introduced to increase interoperability in e-commerce ecosystems that accelerate collaboration in product identification (e.g., GTIN), logistic units (e.g., SSCC), or global data models (e.g., GDM) (GS1 Germany, 2021; Guilloux et al., 2013; Narayanan et al., 2009).

In an e-commerce ecosystem, participants can be attracted by reducing barriers to entry on various levels (Porter, 1980), employing governance decisions to open the focal platform (Boudreau, 2010; Hein et al., 2020), and implementing sophisticated boundary resources for participants to plug in (Aulkemeier et al., 2016a; Eaton et al., 2015; Ghazawneh & Henfridsson, 2013). The provision of platform boundary resources, such as application programming interfaces (APIs) or software development kits (SDKs), involves external application developers focusing on a product, standard system architecture, or communication protocol (Dal Bianco et al., 2014). Boundary resources form an indispensable part of a focal platform’s architecture, facilitating the participant’s interrelation with the periphery as a stable part (Baldwin & Woodard, 2009; Staykova & Damsgaard, 2015).

Ghazawneh (2012) has taken the concept of boundary objects (i.e., repositories, ideal types, coincident boundaries, and standardized forms) from social science and applied it to (software) ecosystems (Ghazawneh, 2012; Ghazawneh & Henfridsson, 2013; Star & Griesemer, 1989). Based on this elicitation, Dal Bianco et al. (2014) distinguished among application (e.g., APIs), development (e.g., SDKs), and social boundary resources (e.g., documentation, developer portals). Acting as a keystone, the platform owner can actively control the boundary resources as part of the platform governance (Hein et al., 2016; Hein et al., 2020; Iansiti & Levien, 2004). Defining the boundaries between the platform owner and the community of third-party developers and other participants facilitates the realization of strategically relevant decisions about ownership, entry into new markets, and community building (Dal Bianco et al., 2014; Foerderer et al., 2019; Hein et al., 2020). Although approaches to strategically develop boundary resources have been proposed in recent research studies (Hein et al., 2019; Star, 2010), standardization of these resources within and across ecosystems has not been considered. Standardized boundary resources are likely to attract additional participants, allow multi-homing, increase the size of the network, and propel network effects (Eaton et al., 2015). Such boundary resources also allow for increased interoperability, modularization, and specialization within an ecosystem and guarantee that complements use boundary resources appropriately (Teece, 2018). The success of platform ecosystems (e.g., Apple iOS, SAP ERP) relies on the provision of sophisticated boundaries for third-party participants that provide additional modules and co-create value for end customers (Eaton et al., 2015; Ghazawneh & Henfridsson, 2010; Hütsch & Wulfert, 2022).

However, for developers providing additional modules to ecosystems, standards do not exist for the application layer in e-commerce ecosystems other than technological, industry-independent, de facto standards such as programming languages or development tools. Thus, the setup time for developers entering a new e-commerce ecosystem is evident, and multi-homing is only possible with additional efforts. In research, the topic of ecosystems, in general, is of increasing interest (Bogers et al., 2019), but little attention has been paid to the technological level and boundary resources, in particular those enabling the interconnection of autonomous participants (Aulkemeier et al., 2016b). Existing design principles in e-commerce focus on user interface (UI) design (Billewar et al., 2021; Foglie et al., 2010; Resnick & Sanchez, 2004; Zollet, 2014), specific technical boundary resources (Sarkar et al., 2007; Verborgh & Dumontier, 2018), or platforms in service networks (Blaschke et al., 2019b) and omit the ecosystem perspective. We explicitly focus on the standardization of boundary resources for developers to ease developers’ work in e-commerce ecosystems, increase the number of developers and complements, and thus propel network effects. We address the problem of proprietary boundary resources in e-commerce ecosystems and propose a set of standardized boundary resources to platform owners to ease the participation of developers and increase the ecosystems’ generativity. Hence, our research question is as follows:

- How should the standardization of boundary resources in e-commerce ecosystems be guided to foster network effects?

To answer our research question, we conducted a systematic literature review and report on multiple case studies extracting prescriptive design knowledge in the form of design principles. Developing prescriptive design knowledge in multiple subsequent case studies that enable reflection of successful designs is an established strategy in information systems research (Gregor & Jones, 2007; van Aken, 2004). Consequently, design principles offer a suitable mechanism to learn from successful designs of the past (i.e., those cases we have selected) and go “beyond a single success story” to “inform other design endeavors” (Chandra Kruse & Seidel, 2017, p. 186). This strategy is paramount to finding cross-case patterns for successful designs and reflections of design principles as part of the theory for design and action (Eisenhardt, 1989; Gregor, 2006; van Aken, 2004). We regard the formulation of design principles as highly relevant, given that we can codify design knowledge about ecosystem design in e-commerce and make it reusable, so that it can be used by others at a different time and reduce the subsequent iterations needed to achieve a successful design (McAdams, 2003). We propose design principles to achieve the standardization of boundary resources in e-commerce ecosystems to reduce barriers to entry for developers and propel network effects. As Reuver et al. (2018) have called for domain- specific conceptualizations of the focal platform, we focus on boundary resources as the transition zones between the core and the periphery of an e-commerce ecosystem (Staykova & Damsgaard, 2015). Consequently, we develop our design principles focusing on the specific requirements of developers within e-commerce ecosystems that extend the focal platform with additional modules (e.g., shop themes, plug-ins, and integration components).

The remainder of this article proceeds as follows: first, we unfold related literature on e-commerce ecosystems as a specialization of digital business ecosystems and the concept of boundary resources in digital platforms. Second, we present our scientific approach of developing design principles to increase the standardization of boundary resources. Third, we elicit our six meta requirements with 19 related design principles. Fourth, we discuss our results, and fifth, we conclude with a short summary and research outlook.

## Related literature

### E-commerce ecosystems

The ecosystem concept was introduced into the business literature by Moore (1993, 1996) as an analogy to biology, in which individual organisms form a population and interact in a habitat. A business ecosystem is formed by independent (or even competing) actors (natural or legal entities), who share a common interest, such as the success of the ecosystem (Corallo et al., 2007; McIntyre & Srinivasan, 2017; Wareham et al., 2014), and who depend on each other (Iansiti & Levien, 2004) and even co-create value (Blaschke & Brosius, 2018). The perspective of business ecosystems is grounded in organizational boundary theory (Santos & Eisenhardt, 2005; Teece, 2007; Tsujimoto et al., 2018). An ecosystem dynamically evolves and is subject to continuous change, as individual participants join or depart while creating new or interrupting existing relationships (Corallo et al., 2007; Wulfert et al., 2021).

The relationships and affiliations among the participants in digital business ecosystems are maintained via information technology (IT), and value co-creation is realized digitally (Tsujimoto et al., 2018). The participants in digital business ecosystems are interdependent. They cooperate to achieve common objectives, usually at the same time competing for scarce resources (Corallo et al., 2007). Thus, a digital business ecosystem is a complex network of platform-mediated, actor-to-actor interactions, becoming increasingly accessible to end users through third parties’ platform complements (Corallo et al., 2007; Wareham et al., 2014). An e-commerce ecosystem is a manifestation of a digital business ecosystem in the context of e-commerce. The value of an e-commerce ecosystem increases for every actor with the addition of each actor in the network (Briscoe et al., 2006; Shapiro & Varian, 1998). Although an increasing number of ecosystem participants amplifies inter-ecosystem competition and diminishes exclusive offers, the overall ecosystem value for each participant on the same and different connected markets is increasing (Zhao et al., 2020). For instance, manufacturers offering similar products within an e-commerce ecosystem compete for similar end customers. The additional ecosystem value is driven by direct and indirect network effects,^1^ increasing the value of participation in a network by actors of both the same and different types (Hinz et al., 2020; Shapiro & Varian, 1998). While this affiliation perspective is mainly concerned with the relationships between the individual actors in an e-commerce ecosystem, the structural view of an ecosystem focuses on the overarching purpose of the ecosystem and its value proposition for a focal company’s end customers (Adner, 2017). The overarching purpose of e-commerce ecosystems is to exchange physical or digital products via electronic communication media (Adner, 2017; Becker & Schütte, 2004; Laudon & Traver, 2020).

E-commerce ecosystems can be formed around a focal platform that can serve several purposes (i.e., innovation and transaction activities), depending on its type (Evans & Gawer, 2016; Evans & Schmalensee, 2016; Gawer, 2014). The platform supports the focal company’s business model and can be characterized as the “technical core” of the ecosystem (Blaschke et al., 2019a, p. 4). Innovation platforms form the operating systems for online shops, electronic marketplaces, and other business models and provide the application services necessary for executing the business processes and environments for developing external modules (Aulkemeier et al., 2016b; Hanseth & Lyytinen, 2010; Tiwana et al., 2010). While platforms usually exploit economies of scale and scope with increasing efficiency and increased product variety through reusability and reconfiguration of modules or services, they may utilize further economic effects as the center of a broader innovation ecosystem (Cusumano & Gawer, 2002; Gawer, 2009; Rochet & Tirole, 2003). Platform extensions and independent modules developed by third-party developers result in the formation of an innovation ecosystem (Manikas & Hansen, 2013; Messerschmitt & Szyperski, 2003). Innovation ecosystems often include dedicated extension marketplaces responsible for providing developed modules (Jansen et al., 2013).

Digital business ecosystems can be structured by a “set of roles” (Adner, 2017, p. 42), describing a standardized relation between the different participants on an abstract level (Jacobides et al., 2018). Eisenmann et al. (2009) have defined four archetypal roles^2^^2^ in an e-commerce ecosystem: users from the demand and supply sides, platform providers, and platform sponsors. The platform owner (i.e., the provider and sponsor) implements governance mechanisms for the focal platform to control the autonomous ecosystem participants and facilitate s value creation (Hein et al., 2020). We consider platform owners as architects of the surrounding ecosystem, defining an interaction structure with a set of boundary resources (Helfat & Raubitschek, 2018).

**Fig. 1:**
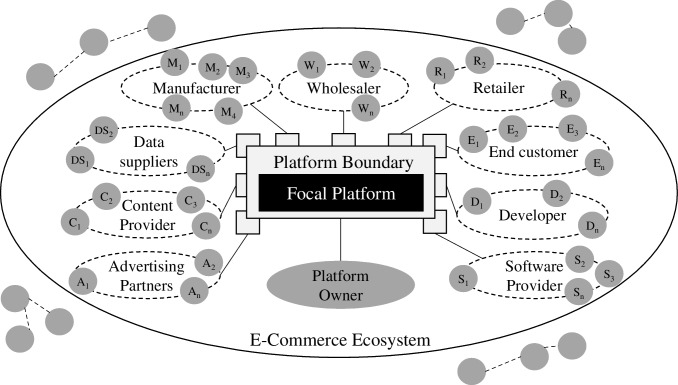
Schematic view of a platform ecosystem in e-commerce with archetypal participants

The major actors in e-commerce ecosystems are the various manufacturers and end customers with intermediating retailers and wholesalers (Böttcher et al., 2021). The original participants mentioned above are augmented by further participants, such as data suppliers, content providers, and advertising partners in e-commerce ecosystems that provide additional information and media in a digital environment (Wulfert & Schütte, 2022). Software providers implement the necessary information systems orchestrating the diverse actors and offer development environments for external developers (Tiwana et al., 2010). Third-party developers may implement additional modules, such as shop themes, interfaces with other digital platforms, or feature add-ins (Wulfert & Schütte, 2021). The participants are connected to the focal platform using a set of boundary resources (Fig. 1). The joint value creation effort of the participating actors in e-commerce ecosystems results in the provision of products and services to end customers (Adner, 2017). An e-commerce ecosystem fulfills the necessary functions, bridging the discrepancies in time, space, and quantity within a supply ecosystem from (raw material) suppliers to end customers (Becker & Schütte, 2004; Laudon & Traver, 2020; Schütte, 2017).

### Boundary resources as theoretical lens

Initially conceptualized in the context of a case study of collaboration among actors with different perspectives but primarily the same objective, boundary objects explain successful collaboration through standardized methods, as well as the development of a set of objects that enable different groups to work together without direct coordination (Star, 2010; Star & Griesemer, 1989). Boundary objects are “plastic enough to adapt to local needs and the constraints of the several parties employing them, yet robust enough to maintain a common identity across sites” (Star & Griesemer, 1989, p. 393). The initial concept from the field of science and research, based on a case study from the Museum of Vertebrate Zoology in California, included four types of boundary resources (i.e., repositories, ideal types, coincidence boundaries, and standardized forms), which did not claim exclusivity and, thus, can be adapted or extended (Star, 2010; Star & Griesemer, 1989). The provision of platform boundary resources enables building a digital business ecosystem involving external application developers around a product, standard system architecture, or communication protocol (Dal Bianco et al., 2014). Even though boundary resources allow access to core modules and integrate developers as participants in the ecosystem, they also act as a control mechanism allowing platform owners to manage the infrastructure based on the strategy pursued, which increases the chances of achieving market leadership (Eaton et al., 2015; Ghazawneh & Henfridsson, 2013).

**Fig. 2:**
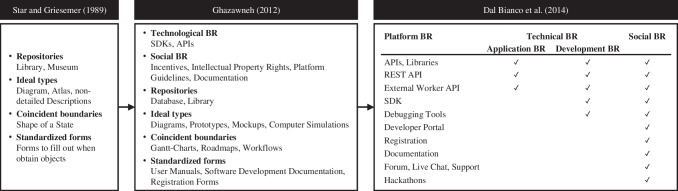
Development of platform boundary resource types (Dal Bianco et al., 2014; Ghazawneh, 2012; Star & Griesemer, 1989)

Designing boundary resources requires considering a variety of different applications. Therefore, it is necessary to find a form that, on the one hand, supports easy software development due to its slenderness and, on the other hand, leaves open creative space so that the innovative ideas of the developer communities can be included in the cultivation of the digital business ecosystem (Dal Bianco et al., 2014; Ghazawneh & Henfridsson, 2013). Boundary resources, as mentioned in the introduction, represent a dimension of platform governance, defining the boundaries between the platform owner and the community of third-party developers, thus facilitating the realization of strategically relevant decisions about ownership, entry into new markets, or community building (Dal Bianco et al., 2014; Foerderer et al., 2019; Hein et al., 2020). In this way, platform boundary resources encompass more than the provision of merely technically relevant resources. Within this research, different types of platform boundary resources that involve both technological and organizational interaction between platform owners and ecosystem participants (e.g., developers) are applied as a theoretical lens to shape e-commerce ecosystem design principles (Dal Bianco et al., 2014; Foerderer et al., 2019; Ghazawneh, 2012). To this end, we draw on theoretical work on platform boundary resources that build on each other (Fig. 2).

Ghazawneh (2012) analyzed how platform owners combine centralized control with decentralized knowledge resource bundles, adding technological and social boundary resources. Dal Bianco et al. (2014) subdivided *technical boundary resources* and designed an onion model in which boundary resource types subsume the properties of other types. In this context, *social boundary resources* are used for knowledge transfer, *development boundary resources *for supporting application development, and *application boundary resources *for enabling interaction with platforms. Application boundary resources (APIs, libraries, etc.) are defined as the minimum required for a digital business ecosystem to be viable. By contrast, development and social boundary resources increase the attractiveness of the ecosystem from the developers’ perspective (Dal Bianco et al., 2014).

Hence, the concept enables transdisciplinary participants in an ecosystem to collaborate and achieve common goals by preserving coherence between overlapping social worlds (Star & Griesemer, 1989; Steger et al., 2018). In this context, boundary objects serve as bridges while having only temporary validity (Star & Griesemer, 1989). Transferring this into the focal platform of an e-commerce ecosystem requires establishing an accompanying process for its standardization, as not all interaction scenarios can and/or should be considered when designing a platform (Hein et al., 2019; Star, 2010). The ongoing adaptation and recreation of boundary resources ensure that the provider exercises infrastructural control while also enabling innovation within an ecosystem (Eaton et al., 2015).

## Methodology

**Fig. 3:**
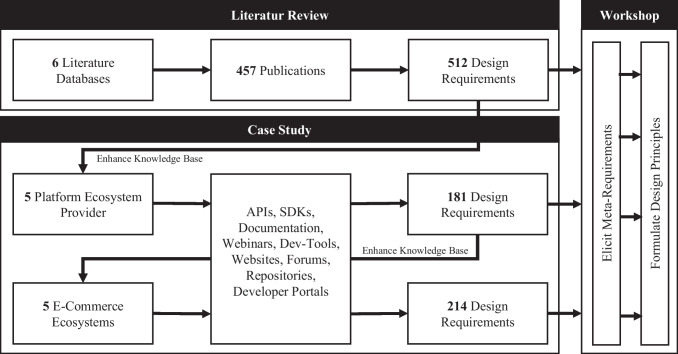
Overall research process

This paper aims to provide a theoretically grounded and empirically validated set of design principles for the standardization of e-commerce ecosystems based on their boundary resources. Design principles are meta-artifacts that belong to the theory for design and action, according to Gregor’s (2006) taxonomy of theory types. Consequently, they are not artifacts per se, but meta-artifacts that enable the codification of design knowledge and make it reusable in different application scenarios (Chandra et al., 2015; Iivari, 2003). Since design principles are *nascent theories*, we devised a strategy to generate design principles from two data sources suitable to develop theory (Gregor & Hevner, 2013; Möller et al., 2020). First, we report on an in-depth systematic literature review, which we used to identify knowledge engraved in the literature corpus and extracted in the shape of *design requirements* (Schryen et al., 2015). Second, we complement these findings with a multiple-case study using comparative analysis to identify relevant design knowledge suitable for abstraction and codification into design principles (Yin, 2014). The dual approach (Fig. 3) that we propose is expressively suitable to generate theory, as the systematic literature review ensures the inclusion of existing knowledge in theory building (Webster & Watson, 2002), and the multi-case study complements it through its high “likelihood of generating novel theory” (Eisenhardt, 1989, p. 546).

Since our view on e-commerce ecosystems is focused on platform boundary resources, the case data and the literature review (i.e., the coding of the papers) are viewed through a theoretical lens (Niederman & March, 2019) that we developed during the elaboration of the theoretical foundation. The theoretical lens is a product of an iterative analysis of the literature conceptualizing platform boundary resources (Appendix 2). Generally, we started with the generic classification of boundary resources into *social boundary resources*, *platform boundary resources*, and *development boundary resources*, following Dal Bianco et al. (2014). By entangling these concepts with more nuanced ones, we constructed a hierarchy of concepts, with the final leaf being the theoretical lens fragment that guided our coding process. For example, we specified *platform boundary resources* to *APIs* and *coincident boundary* (Star & Griesemer, 1989). We detailed the latter more to accommodate our inductive findings, from which we judged that further detailing of the concept was required and sensible (Appendix 2). In the following sections, we describe in more detail how both methods (i.e., the systematic literature review and the multi-case study) intertwine conceptually to formulate design principles.

### Systematic literature review

The first phase aims to analyze the existing literature and the associated deduction of initial design requirements. Based on process and quality criteria, the methodological approach of a systematic literature search offers an appropriate framework to ensure the traceability, systematicity, and reproducibility of the results (Cram et al., 2020). Following this principle, our literature review follows the methodological approach of Webster and Watson (2002), in combination with vom Brocke et al. (2015). The foundation of the search is the conceptualization of the object under consideration (i.e., boundary resources) and its integration into the domain of e-commerce. We combined the relevant boundary resources with the conceptual variety of the domain based on the developed theoretical lens and resulting search terms (Appendix 2). The boundary resources search terms are aggregated in Fig. 6.

**Fig. 4:**
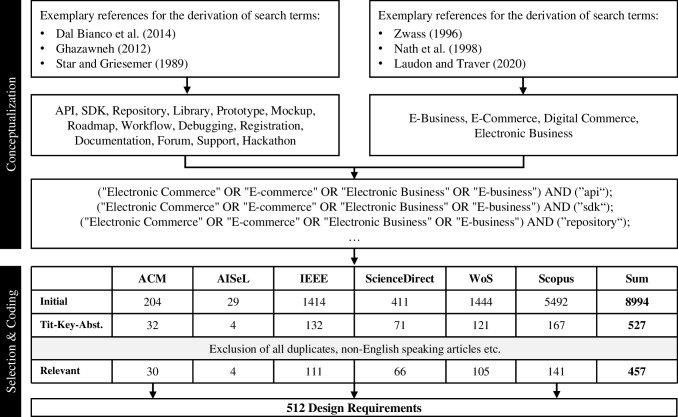
Literature review and coding-process

In total, 84 search strings were performed in six databases, with initially 8,994 hits in April 2021. To ensure an appropriate level of quality, additional quality criteria were added to the search. Excluded were non-English language articles; panels and commentaries; purely technical articles (e.g., articles that focus exclusively on technological aspects without applying them to an e-business platform ecosystem context); and articles with a pure e-business focus (e.g., articles that focus exclusively on e-business or subtypes without adopting a corresponding e-commerce ecosystem perspective). Based on the title and abstract considering the quality criteria, and following the approach of Bandara et al. (2015), 527 relevant publications could be identified within the initial set. After excluding duplicates, 457 publications were defined as the final literature sample. The articles within the sample were independently analyzed using full-text screening, and the relevant text passages were coded to extract the requirements. The 14 conceptualized boundary resources served as the basis for coding, which was inductively adapted as needed. Based on the coding scheme, 512 requirements were extracted in four coding iterations (Fig. 4).

### Multi-case study analysis

To develop design principles that are grounded in empiricism, we report on a multiple-case study (Eisenhardt, 1989; Yin, 2014). Explicitly, we chose a multiple-case study since it enabled us to collect design knowledge more broadly from a series of cases that might differ in environmental aspects but share a common phenomenon (Yin, 2014). Selecting multiple cases is paramount in identifying cross-case patterns and as the basis for comparative analysis (Eisenhardt, 1989; van Aken, 2004). For example, Avdiji et al. (2020) have demonstrated the power of cross-case analysis and the abstraction of design knowledge from that process. In our research, we identified ten cases as the basis to derive design knowledge about thriving e-commerce ecosystems. Having multiple cases is necessary to transcend the specificity of each individual case and abstract generally applicable knowledge that benefits the class of the artifact (Gregor et al., 2013; Lee et al., 2011), that is, standardized e-commerce ecosystems. We structured the multi-case study into two thematic categories to accommodate their shared environment (Yin, 2014). The first set contains five *Platform Ecosystem Providers* **(A)**, cases providing the technical infrastructure of successful e-commerce ecosystems*,* and the second set contains five *E-Business Platform Ecosystems* **(B)**, cases applying a platform business model in e-commerce (Schütte & Wulfert, 2022; Wulfert & Schütte, 2021). While these sets differ in environmental aspects, they enabled the analysis of how ecosystems work in e-commerce, giving us ample opportunity to generate theory from all cases (Table 1). Further details on cases analyzed regarding business model, number of developers, and participant orientation can be found in Appendix 1.

**Table 1:** Key Performance Indicators of Case Platforms

Platform Ecosystem Providers (A)	Shopware	Magento	SAP Commerce Cloud	Saleforce Commerce Cloud	OXID eShop
Establishment	2004	2008	2012*	1999	2003**
Country of origin	Germany	USA	Germany	USA	Germany
Number of employees	300	375	---	49.000	51–200**
Anual revenue (billion euro)	0,02	84,7	---	14,5	---
E-Business Platform Ecosystems (B)	Amazon	Walmart	Ebay	Etsy	Otto
Establishment	1994	1962	1995	2005	1949
Country of origin	USA	USA	USA	USA	Germany
Number of employees	1.298.000	2.200.000	13.300	1.240	49.895
Anual revenue (billion euro)	362,9	473,5	9,1	1,4	15,6

* = Year of publication of the SAP Cloud Platform.

** = Values refer to the OXID eSales AG.

Since we wanted to produce prescriptive design knowledge that enables designers to design e-commerce ecosystems more efficiently and learn from past success stories, we collected design knowledge from highly successful ecosystems in e-commerce (Chandra Kruse et al., 2019). Consequently, the *environment* of each case indicated a high degree of success in establishing highly successful ecosystems in e-commerce. Correspondingly, the *shared phenomenon* that we investigated is how these cases leverage the utility and potential of standardized platform boundary resources. In selecting the cases, we explicitly targeted “large” platform owners with substantial ecosystems. We also tried to include prominent and successful cases from China, such as Alibaba and JD. However, the language barrier proved to be an obstacle too difficult to overcome without a significant loss of comprehension of the underlying data, which is why we opted to select only cases with data available in the German or English languages. In addition, we opted to include only successful cases to codify “successful” knowledge and make it applicable to others (Chandra et al., 2015; Gregor et al., 2020). Subsequently, we excluded Rakuten, given its transition from a marketplace to a cash-back platform and its decrease in global availability. In summary, we selected focal platforms of successful e-commerce ecosystems on the business and infrastructure layer, in terms of size and identity (Cennamo, 2021), that leverage the utility and potential of standardized boundary resources. Ultimately, we identified the following ten cases that we used to reflect and abstract our findings (Gregor, 2009). While category A consists of the cases of Salesforce Commerce Cloud, Magento, SAP Commerce Cloud, Shopware, and OXID eShop, category B contains Amazon marketplace, Walmart marketplace, eBay, Etsy, and Otto (Appendix 1).

We leveraged a plethora of publicly available data sources to collect information on each case. Specifically, we analyzed the case data through the theoretical lens developed above, ensuring that we framed it distinctively in the context of platform boundary resources (Niederman & March, 2019). Naturally, we screened only official information on each case. We did not limit ourselves to the homepages of the companies alone and therefore screened all company sources on different platforms (e.g., GitHub, YouTube). Since we analyzed the data through the lens of platform boundary resources, we included technical documentation on APIs and SDKs as primary data sources. In addition, we looked through available information, codes, documentation, and meta-data on GitHub for each case. Moreover, we analyzed corresponding development tools offered by platform providers for development or prototyping to fully understand the necessary steps to utilize the platform from the ecosystem participants’ perspective. Appendix 1 contains an extract of sources leveraged for each case company selected and includes a matrix providing transparency on the impact of the case companies on our design principles (Table 16).

In the dual approach, we extracted detailed design requirements that stayed on a very narrow conceptual level and close to the wording and content of the underlying literature and/or case data (Figure 4). The procedure resulted in an accumulated set of 900 design requirements from the literature and case analysis (Figure 3). Since design principles require addressing a class of artifacts rather than an instance, we correspondingly elevated the set of design requirements to a higher order of meta-requirements (MRs) (Walls et al., 1992). In addition, developing design principles based on meta-requirements ensures *value grounding*, meaning that no design principles exist without fulfilling at least one requirement (Goldkuhl, 2004). We did this by, first, excluding duplicates from the design requirements as well as those we identified as not being crucial. In a second step, following the recommendations of Koppenhagen et al. (2012), we used logical content aggregation in a series of workshops by the author team to distill the most relevant requirements of the class of artifact, that is, meta-requirements (Thoring et al., 2020). Based on that aggregation process, we generated six meta-requirements for designing boundary resources in e-commerce ecosystems to guide our formulation of design principles (Möller et al., 2020).

## Formulation of design principles

This section reports on the meta-requirements and design principles for standardized boundary resources in e-commerce ecosystems that we developed based on the literature corpus and the multi-case study. In the following, we introduce the six aggregated and condensed meta-requirements, alongside a short explanation (Table 2). The first meta-requirement (MR1) addresses the need to *tailor boundary resources* to accommodate the particular needs of different ecosystem participant types in terms of access to the ecosystems and communication levels. MR2 addresses the need for *openness for the ecosystem*, expressed in an open architecture or the possibility to contribute third-party applications. MR3 addresses issues of *trust and risk*, demanding that boundary resources in e-commerce ecosystems implement mechanisms for security when selecting, for example, third-party modules or supplies on marketplaces to lower the chance of fraud. MR4 requires that these ecosystems provide a minimal shared understanding of institutionalized *rules and standards* to enhance the quality of components and streamline integration into the ecosystem. MR5 (*development environment*) requires the provision of streamlined workflows for developers and standardized mechanisms to contribute third-party modules (e.g., integrated development environments or demos). Last, MR6 requires the *curation of the ecosystem* by a platform owner through providing documentation and good practices in early communication of changes to the codebase to enhance reliability for developers. Table 2 summarizes the meta-requirements with a corresponding short title and using the modal verb “should” (Offermann et al., 2010).

**Table 2:** Meta-requirements for designing boundary resources in e-commerce ecosystems

#	Short Title	Meta-Requirement
MR1	Tailored Boundary Resources	Boundary resources in e-commerce ecosystems should be tailored to the requirements of ecosystem participants
MR2	Openness of the Ecosystem	Boundary resources in e-commerce ecosystems should foster the openness of the ecosystem and use an open architecture
MR3	Trust and Risk	Boundary resources in e-commerce ecosystems should implement mechanisms to enhance trust and security when selecting third-party modules
MR4	Rules and Standards	Boundary resources in e-commerce ecosystems should institutionalize a minimal common understanding of rules and standards for participants
MR5	Development Environment	Boundary resources in e-commerce ecosystems should provide developers with streamlined workflows to contribute third-party modules
MR6	Ecosystem Curation	Boundary resources in e-commerce ecosystems should actively curate the ecosystem through mechanisms such as documentation and communication

**Fig. 5:**
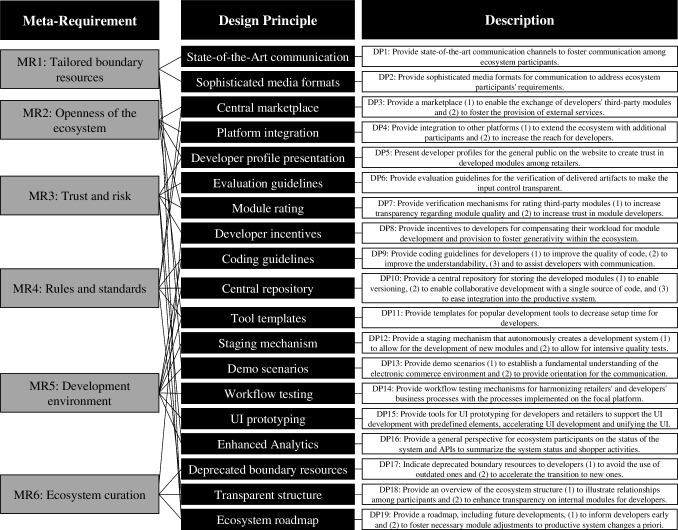
Meta-requirements and design principles for boundary resources in e-commerce ecosystems

**Fig. 6:**
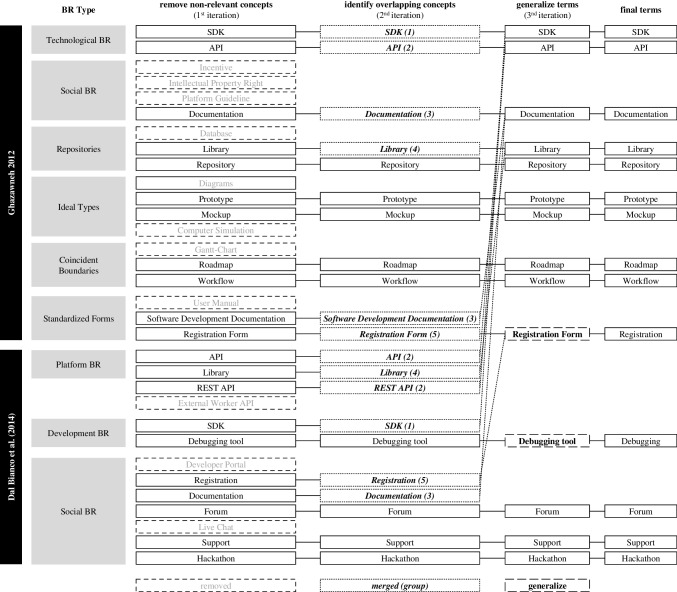
Iterative process for deriving keywords based on the literature

Figure 5 links the six meta-requirements above with 19 design principles that we explain in detail below. The mapping diagram depicts the fulfillment of requirements by the design principles, with links between them, and provides each design principle with a short textual description (Möller et al., 2020).

### Tailored boundary resources

- **DP1:** Provide state-of-the-art communication channels to foster communication among ecosystem participants.

#### Rationale

The establishment and maintenance of relationships among ecosystem participants rest on synchronous and asynchronous communication. This communication can be fostered by state-of-the-art communication channels, such as developer forums to exchange ideas and discuss problems (Magento, 2021f; Sap SE, 2021c), blogs to inform developers about future developments and provide developers with a platform to present their new projects and solutions (Liang et al., 2011; Otto SE, 2021c), and an online code editor with syntax highlighting for collaboration (salesforce Inc., 2021e). In addition to these, the platform owner can implement a dedicated support system (Ahmed, 2020), offer a live chat bot that answers questions (Elmorshidy, 2011; Li et al., 2017), and present frequently asked questions and solutions for already-recognized issues (Büttner et al., 2019). These channels should create and foster social interactions to satisfy developers’ social needs for belonging and support (Liang et al., 2011, p. 70). Social interaction can also be addressed by hosting hackathons to create new solutions for the e-commerce ecosystems (Otto SE, 2021a).

- **DP2:** Provide sophisticated media formats for communication to address ecosystem participants’ requirements.

#### Rationale

As developers are habituated to sophisticated media formats because of prior personal and professional experiences, boundary resources using sophisticated media formats should be presented by the platform owner. This will increase the overall communication quality with ecosystem participants in general and developers in particular. The platform owner can offer online collaboration with synchronized editing, voice messaging, web conferencing, messaging, and video streaming to communicate boundary resources to developers (Sokiyna & Aqel, 2020). Our case study analysis revealed that interactive webinars with gamification elements and micro learning are often offered to engage developers in training (salesforce Inc., 2021d; Sap SE, 2021d). Furthermore, platform providers organize online keynotes and virtual face-to-face meetings with developers (salesforce Inc., 2021a). In addition to the application of media formats, all platform cases analyzed provide extensive textual descriptions. This is especially relevant for documentation on technical boundary resources provided to developers (Etsy, 2021a; GitHub, 2021; Otto SE, 2021b).

### Openness of the ecosystem

- **DP3:** Provide a marketplace (1) to enable the exchange of developers’ third-party modules and (2) to foster the provision of external services.

#### Rationale

A central marketplace for providing third-party modules and external services (e.g., address validation, logistics services, content provision), integrated into the focal platform and provided by the platform owner, can encourage the intention to use these modules and services among ecosystem participants (Hajli, 2013). The platform owner as an intermediary increases trust in external modules and services (Verhagen et al., 2006). The analysis of our case studies demonstrated that there is no consensus established to offer a dedicated store for modules to be installed and a separate one for services or a store including both types. While Salesforce provides a store for external modules (i.e., app exchange) and a store for services provided by partners (i.e., partner marketplace) (salesforce Inc., 2021b, 2021c), SAP integrates both types within a single store, Magento offers additional modules, and Walmart only provides self-developed, ready-to-use modules without a dedicated store (Magento, 2021a; Sap SE, 2021b; Walmart Corp., 2021a).

- **DP4:** Provide integration to other platforms (1) to extend the ecosystem with additional participants and (2) to increase the reach for developers.

#### Rationale

An existing e-commerce ecosystem can be extended by connecting the focal platform with its provided services and products to other platforms and their surrounding (e-commerce) ecosystems. This increases the dissemination of the focal platform and enables developers to reach additional participants of other ecosystems. Piggybacking on an existing ecosystem is a well-known strategy for establishing and disseminating platforms (e.g., PayPal using eBay to attract participants) in electronic business (Evans & Schmalensee, 2016; Parker & van Alstyne, 2016). To establish a relation and implement a connection between two or more platforms, integrations services, connectors, protocol conversion, or message transformation are required. This integration ensures a seamless flow between the platforms without any interruption for developers or customers. Moreover, the focal platform may optimize its pluggability for other platforms to increase the generativity and variety of the ecosystem and to attract additional participants, increasing network effects (Aulkemeier et al., 2016a). Integration services to other electronic marketplaces, shops, or payment platforms are often deployed via stores providing external modules (Magento, 2021b; Oxid AG, 2021b). Software vendors such as Salesforce or SAP integrate their shopping systems with their portfolio of applications.

### Trust and risk

- **DP5:** Present developer profiles for the general public on the website to create trust in developed modules among retailers.

#### Rationale

The developer profiles help clarify which independent or certified developer has contributed to the e-commerce ecosystem. This transparency increases trust in external modules (Teubner & Dann, 2018). Providing detailed profiles, including portraits of the developers, creates relationships on a social level (Hajli, 2013). The publication via the focal platform, as an intermediary between developers and users of the modules, increases the established trust (Tan et al., 2015). At Magento, for example, the number of created and accepted contributions of a developer is published. There is also a scoring system to determine the contributors of the month, the quarter, and the year (Magento, 2021g).

- **DP6:** Provide evaluation guidelines for the verification of delivered artifacts to make the input control transparent.

#### Rationale

It should be transparent which evaluation guidelines have to be followed by the developers to comply with the guidelines determined by the platform owner and the requirements of the platform (Hein et al., 2016; Tiwana et al., 2010). In general, these guidelines improve the quality of external modules and make quality ratings transparent for developers and users of the modules. These guidelines can even be transferred to other platforms using platform-independent criteria (Hesse et al., 2020; Teubner & Dann, 2018). At Etsy, approval review criteria have to be reviewed before the artifact is released to the public (e.g., applications must not sidestep the API to retrieve or post Etsy data) (Etsy, 2021a).

- **DP7:** Provide verification mechanisms for rating third-party modules (1) to increase transparency regarding module quality and (2) to increase trust in module developers.

#### Rationale

To further strengthen the trust in third-party modules, module ratings can be used. The module ratings represent the trustworthiness and usefulness from the user perspective. This helps the developer get feedback from the users and improve the quality of their modules (Dalvi et al., 2016). In addition, the third-party modules can be awarded gold, silver, and bronze ratings for their quality and support from the perspective of the platform provider. Metrics can include how many times a module has been used, how many ratings have been given, how good the ratings were, how many updates there have been to the module, how long the developer has been registered on the platform, and how many total extensions the developer has created (Shopware, 2021c). Another aspect is that the developers must be registered and often authorized to publish their modules (e.g., at the Amazon Marketplace developers receive their own IDs).

- **DP8:** Provide incentives to developers for compensating their workload for module development and provision to foster generativity within the ecosystem.

#### Rationale

While the relationships between the focal platform and its customers play a significant role in social commerce, this can also be applied to the relationship between the platform and the developer (Liang et al., 2011). Rewards and incentives should be introduced to stimulate developers’ generativity and engage them to contribute to e-commerce ecosystems with additional modules. In general, incentives can increase the loyalty of developers and are often offered on a monetary basis. However, incentive plans should not only be influenced by the number of modules provided and their reach among ecosystem participants, but also by their overall quality. Receiving a reward can also be seen as a social incentive (Magento, 2021g).

### Rules and standards for module development

- **DP9:** Provide coding guidelines for developers (1) to improve the quality of code, (2) to improve the understandability, (3) and to assist developers with communication.

#### Rationale

The use of coding guidelines has already been proven in open source projects (Szyjewski, 2019). It becomes a prerequisite in an ecosystem where participants with different perspectives and no direct communication need to coordinate. This might include guidelines for programming languages, stylesheet languages, scripting languages, markup languages, or the interplay between languages at different architectural levels. Compliance with coding guidelines should be supported by offering utilities that automatically adjust the code or offer live support to developers within the development environment. The coding guidelines can be based on established standards, such as the PSR family for developing extensions in PHP, which provide a syntactic and semantic framework for code design (Szanto, 2021). Tools such as ESLint or Standard JS can be used in JavaScript to automatically check compliance with coding guidelines and adjust them if needed.

- **DP10:** Provide a central repository for storing the developed modules (1) to enable versioning, (2) to enable collaborative development with a single source of code, and (3) to ease integration into the productive system.

#### Rationale

Collaboration among ecosystem participants should be supported by an easily accessible, central repository containing the status of available e-commerce ecosystem modules. This enables coordination of projects as well as suggestions for features or bug fixes from the community. Using a central repository further increases the activity level of individual ecosystem projects and enables goals to be achieved faster (Alshomali et al., 2017). In this context, the version control system Git, enabling distributed and transparent tracking of code changes, emerged as a de facto standard (Govil et al., 2020). Cloud-based services combine version control with free hosting space, as well as social features, making them ideal for collaboration among different participants in an e-commerce ecosystem (Alshomali et al., 2017). By offering their boundary resources, they also enable deployment processes to be established, positively affecting system integration in the long term (Wei et al., 2020).

- **DP11:** Provide templates for popular development tools to decrease setup time for developers.

#### Rationale

Establishing the conditions for easy and rapid development of new services around the focal platform further increases the opportunity for more third-party developers appreciating and expanding the e-commerce ecosystem. The setup effort for potential third-party developers can be reduced by providing templates and instructions for the optimal configuration of development tools. This includes the provision of pre-sets for popular integrated development environments, settings for utilities, and the configuration of local development web servers (Magento, 2021h; Oxid AG, 2021a; Shopware, 2021a). Furthermore, customized plug-ins and extensions for integrated development environments or browsers can noticeably shorten the initial training period (salesforce Inc., 2021g, 2021h).

### Development environment

- **DP12:** Provide a staging mechanism that autonomously creates a development system (1) to allow for the development of new modules and (2) to allow for intensive quality tests.

#### Rationale

The development environment and the productive environment should be very similar but separate from each other. Before new modules are released in the productive environment, they should be extensively tested for their functionalities in a development system that contains sample data. Sandbox environments can be used for this purpose. In the sandbox, test users can be created who can act as sellers or customers (eBay, 2021b). Orders, shipments, and returns can be simulated to test API connections with the focal platform (Otto SE, 2021b; Walmart Corp., 2021c). After intensive testing, the new module should be smoothly transferred to the productive environment of the focal platform.

- **DP13:** Provide demo scenarios (1) to establish a fundamental understanding of the electronic commerce environment and (2) to provide orientation for the communication.

#### Rationale

As the possibilities in e-commerce are manifold, the developers should familiarize themselves with implementation opportunities. Demo scenarios can be a helpful starting point because they can also identify unforeseen opportunities and obstacles (Svátek et al., 2015). Various demos have been offered at Magento. Business-to-business (B2B) demos, front-end demos, back-end demos, and administration demos are available. The SAP Customer Experience Lab offers numerous showcases for the SAP Commerce Cloud (Sap SE, 2021e). In this way, communication with the provider or between developers can be supported.

- **DP14:** Provide workflow testing mechanisms for harmonizing retailers’ and developers’ business processes with the processes implemented on the focal platform.

#### Rationale

The business workflow must run correctly in e-commerce ecosystems; if it does not, significant financial damage can occur. It is still a considerable challenge for developers to design, analyze, control, and diagnose a business workflow due to the complexity of the e-commerce environment (Wu & Lin, 2011). To support this, the workflow should be visualized transparently. Templates can be used to represent different situations and to help the user understand the meaning of the business process more quickly (Wu & Lin, 2011). Appropriate modeling languages should then be used accordingly. For example, commonly used workflow models are based on active networks, on event-driven process chains, on speech act theory, on Petrinets, and on Unified Modeling Language (Cai, 2012). In addition to understanding and representation, the possibility should be provided for the workflow to be tested. It is imperative to verify and validate the functionalities, especially if new functionalities have been implemented (Walunj & Sadafale, 2013).

- **DP15:** Provide tools for UI prototyping for developers and retailers to support the UI development with predefined elements, accelerating UI development and unifying the UI.

#### Rationale

The development of a prototype generally indicates the feasibility of a business process (Aulkemeier et al., 2016a). In the context of UI, the prototype can also be employed to assess the usability of the e-commerce website (Aulkemeier et al., 2019). Users can be observed and questioned afterward, for example on their impressions, complaints, and suggestions (Mushthofa et al., 2018). To avoid every third-party developer having to observe the user again, the platform provider should provide predefined and evaluated elements. It also helps to ensure that the elements are unifying. Magento, for example, even presents the UI components in detail in the documentation for the developers.

- **DP16:** Provide a general perspective for ecosystem participants on the status of the system and APIs to summarize the system status and shopper activities.

#### Rationale

In e-commerce, it is becoming increasingly important to address customers individually and provide them with suitable and customized offers (Da Silveira et al., 2001). For this, tools must be available that customers understand in real time, for example, during navigation (Marcondes et al., 2017). Customers can in future also use big data to find an e-commerce website that suits them individually (Malhotra & Rishi, 2021). One step below is the analysis for errors in the system, for example, in the case of interface errors. Further steps can be triggered automatically (Marcondes et al., 2017). In addition, different ecosystem participants need the analysis to be accessible to non-experts (Diamantini et al., 2015). Therefore, enhanced analytics are recommended. On this basis, further steps can then be derived to influence shopper activities positively in economic terms.

### Ecosystem curation

- **DP17:** Indicate deprecated boundary resources to developers (1) to avoid the use of outdated ones and (2) to accelerate the transition to new ones.

#### Rationale

An e-commerce ecosystem must evolve to be sustainable because of strategic decisions and technological developments (Wareham et al., 2014). This involves the evolution of the focal platform as the core of the ecosystem and external modules (periphery), while the boundary resources must be relatively stable (Baldwin & Woodard, 2009; Staykova & Damsgaard, 2015). Nevertheless, the boundary resources must also be updated over time, creating new versions while abandoning deprecated ones. Hence, the principle of deprecated boundary resources should ensure that deprecated resources are appropriately handled by the platform owner when new resources are designed, and that proper versioning of boundary resources is established. Deprecated boundary resources should be announced a priori with an appropriate lead time, and transition paths should be demonstrated (Ghanam et al., 2012; Magento, 2021d). While our case study analysis demonstrated that most of the platforms indicate deprecated boundary resources (eBay, 2021a; Etsy, 2021b; Shopware, 2021b), transition plans are seldom employed, often leaving developers with the task of analyzing changelogs on their own. In addition, the stability of boundary resources should be guaranteed, as exemplified by Salesforce’s three-year plan from their first launch (salesforce Inc., 2021f).

- **DP18:** Provide an overview of the ecosystem structure (1) to illustrate relationships among participants and (2) to enhance transparency on internal modules for developers.

#### Rationale

Focal platforms in e-commerce ecosystems comprise several interdependent and interacting components (Aulkemeier et al., 2019; Yu & Ni, 2013). Especially in agile e-commerce projects documentation quality is often neglected, resulting in insufficient transparency concerning the overall system architecture (Sensuse et al., 2020). However, the expansion of core e-commerce functionalities leads to challenges in integrating new services into the core system (Aulkemeier et al., 2016a). Therefore, platform providers need to provide appropriate social boundary resources that document a platform’s present architecture and clarify the interrelationships of internal services with application boundary resources. The documentation should also explain key underlying architectural principles (e.g., command query responsibility segregation) (Magento, 2021c).

- **DP19:** Provide a system roadmap, including future developments, (1) to inform developers early and (2) to foster necessary module adjustments to productive system changes a priori.

#### Rationale

The evolution of the focal platform envelops additional features and addresses further markets, as the core of an e-commerce ecosystem affects third-party developers if previously stable boundary resources are adapted (Eisenmann et al., 2011; Hermes et al., 2020). Platform owners can present roadmaps for the communication of additional platform features and forthcoming boundary resource adjustments so that developers can leverage new features within their existing and new modules. Roadmaps enable developers to plan necessary adjustments and keep modules operational (Ghanam et al., 2012). Our case study analysis demonstrates that platform ecosystem providers communicate significantly more future platform adjustments than e-business platform owners. While Magento releases new software versions quarterly with a priori announcements and SAP offers a detailed web-based roadmap explorer and management summary (Magento, 2021e; Sap SE, 2021a), Etsy only provides an abstract roadmap (Etsy, 2021a). Walmart does not provide exact dates for developments and only randomly announces future developments via the “what’s new” section of the developer blog (Walmart Corp., 2021b).

## Discussion

This section presents a preliminary evaluation, followed by the paper’s scientific and practical contribution, and the limitations of this research. Focal platforms form the core of e-commerce ecosystems. While standards already exist on several levels (e.g., process harmonization across a supply chain and interface standards such as EDIFACT) for e-commerce ecosystems, the relation between the focal platform and external developers lacks standardization, in addition to de facto standards for software providers and implementation guidelines. Our 19 design principles were developed following the second strategy proposed by Iivari (2015), generalizing from ten specific platform ecosystems in e-commerce, supported by an extensive literature review. As prescriptive statements, design principles are reusable for a multitude of instances of a system class (Kuechler & Vaishnavi, 2008; Venable, 2006). Thus, we follow the framewor for “light reusability evaluation of design principles” (Iivari et al., 2021, p. 291), along with the criteria accessibility, importance, novelty and insightfulness, actability and guidance, and effectiveness, as suggested by Möller et al. (2020), for the evaluation of our design principles. Our *preliminary evaluation* was executed analytically (Jenkins, 1985; Väyrynen & Iivari, 2015). As design principles for a class of systems form a unit of prescriptive knowledge, they should be evaluated as a comprehensive set (Iivari et al., 2021).

Our design principles can be applied by platform owners (i.e., software providers, e-commerce companies) in e-commerce ecosystems. The class of systems under consideration (i.e., focal platforms) is apparent to practitioners. The design principles are *accessible*, as we apply a language known to domain experts. We utilized the framework suggested by Chandra et al. (2015), resulting in a structured design principle formulation and, based on this, we provide a detailed set of 19 design principles for a holistic standardization of the focal platform, focusing on developers. Our design principles are *important* to practitioners, as the significance of platforms in dynamic e-commerce ecosystems increases and platforms penetrate almost every sector (Choudary, 2015; Evans & Schmalensee, 2016; Wulfert et al., 2021). In addition, platforms are introduced to augment physical products, with additional smart services fostering a hybridization of products. A comprehensive set of 19 design principles focusing on the standardization of e-commerce ecosystems for developers is a *novel* approach. Our theoretical lens of boundary objects can provide new *insights* for ecosystem control, curation, and development. Our design principles provide immediate utility, as they are *actable* and directly implementable for platform owners, with exemplary design instances (Kuechler & Vaishnavi, 2011). Although we provide *guidance,* including design instances for several design principles, platform owners can be flexible regarding the specific implementation of the prescriptions. Platform owners can decide on the selection of boundary resources and add others to best support their specific e-commerce ecosystems. Thus, we achieved a reasonable balance between guidance and flexibility (Chandra Kruse et al., 2016). The design principles can be *effectively* reused by the owner of a focal platform in an e-commerce ecosystem on different (architectural) levels. We tailored the design principles for actor, business, and application level, using our theoretical lens. The design principles (positively) affect the development of third-party modules by accelerating the overall degree of standardization within and across ecosystems.

As *scientific contribution*, we provide a novel set of 19 design principles for a class of information systems (i.e., focal platforms in e-commerce ecosystems). These design principles guide the relation between platform owners and third-party developers by standardizing boundary resources across e-commerce ecosystems (Hein et al., 2020). Design principles for boundary resources in e-commerce ecosystems specialize the general research on boundary resources by Star and Griesemer (1989) and Ghazawneh (2012). We emphasized the role of boundary resources for the proliferation of e-commerce ecosystems and their role in propelling network effects to reach a minimum threshold of ecosystem participants (Eisenmann et al., 2009; Parker et al., 2016). We also proposed a differentiation of focal platforms in e-commerce ecosystems (i.e., platform ecosystem providers, e-business platform ecosystems) in line with Gawer (2021).

Although we propose design principles for the standardization of e-commerce ecosystems with a focus on boundary resources, for the *practical contribution* it needs to be discussed which ecosystem actors have the power to set (technical) standards within and across e-commerce ecosystems (Iansiti & Levien, 2004). The owners of a focal platform can promote ecosystem-wide standards as part of their governance function, but only have a minor interest in standardization across e-commerce ecosystems (Cennamo, 2021), as cross-ecosystem standardization may enable multi-homing of participants. Cennamo (2021) has argued that platforms with an established base of participants are likely to increase barriers to entry (e.g., by proprietary boundary resources) to lock in ecosystem participants and increase the quality of extensions developed externally, requiring registration fees, extensive certifications, and quality checks for modules. With its focal platform, Amazon is already setting proprietary standards for product stocking and shipping requirements, differentiating itself from other ecosystems, to lock in ecosystem participants (Jacobides et al., 2018). Independent software vendors that offer a software platform for e-commerce (e.g., Magento, Shopware) can also act as the standard-setting organizations, as defined by Viardot et al. (2021).

The derivation of design principles for boundary resources in e-commerce ecosystems indicated a tendency for software vendors to focus on the development of boundary resources for third-party developers, while e-business platforms are more concerned with platform boundary resources linking supply-side participants. Social boundary resources are employed equally by the case companies selected. In contrast to platform owners, developers significantly profit from the standardization of ecosystems, enabling multi-homing and increasing the dissemination of their modules. The increased reach of developers can also be desirable for other ecosystem participants, such as content providers and suppliers (GitHub, 2021). These participants require a different set of design principles for standardization, as they are less interested in developing modules and more interested in business processes and social boundary resources.

Based on our multi-case study results, we also took an internal platform perspective, investigating possible responsible agents for implementing or benefitting from the design principles. We allocated our 19 design principles to responsible agents at focal platforms in e-commerce ecosystems (i.e., business process owner, user interface designer, internal platform development, and infrastructure provision). The roles were derived based on the layers of digital platforms, as proposed by Zutshi and Grilo (2019). The mapping in Table 3 indicates the potential relevance of each design principle for the four roles. Overall, the mapping indicates that internal platform development (3), responsible for implementing APIs and SDKs for third-party developers, is concerned with most of the design principles. While business process owners (1) are responsible for integrating third-party development at the business level, user interface designers (2) should ensure a standardized and uniform user interface across modules. The infrastructure provision (4) ensures flexible computing power for the focal platform and is also involved in ecosystem orchestration.

**Table 3:** Association of design principles to roles in the focal platform

Role in the Platform	Design Principle
(1) Business Process Owner	DP1, DP2, DP3, DP4, DP5, DP13, DP14, DP16, DP18, DP19
(2) User Interface Design	DP1, DP2, DP4, DP9, DP10, DP11, DP13, DP15
(3) Internal Platform Development	DP1, DP2, DP3, DP5, DP8, DP9, DP10, DP11, DP12, DP13, DP14, DP16, DP17, DP18, DP19
(4) Infrastructure Provision	DP5, DP6, DP7, DP8, DP9, DP12, DP13, DP16, DP17, DP18, DP19

When applying our design principles, platform owners need to carefully balance the standardization of boundary resources without limiting developers’ flexibility and freedom in pursuing their ideas. Excessive standardization may contradict developers’ generativity and, thus, reduce the overall value proposition for customers (Adner, 2017). Standardization of boundary resources in e-commerce ecosystems to attract additional participants is a particular trade-off in favor of the generativity of developers. We argue that a higher degree of standardization of boundary resources will enhance network effects and, as a result, the overall value of the ecosystem for participants. Opening the focal platform, offering access to additional sections of the platform core, and integrating with other platforms may attract additional developers; however, the pursuit of openness can reduce a focal platform provider’s control over the e-commerce ecosystem (Hein et al., 2020).

Star (2010) and Hein et al. (2019) have emphasized that the definition of standards is never complete. Rather, it is a continuous process deriving residual categories, developing boundary objects, and updating or creating new standards. We have analyzed a snapshot of the boundary resources of our ten cases for one point in time. As e-commerce ecosystems and their focal platforms, including boundary resources for participants, are subject to dynamic evolution (Wulfert et al., 2021), our design principles may also require an update in the future. Therefore, a process for standardizing boundary resources can be established in analogy to Hein et al. (2019). The management of external developers and boundary resources is a crucial activity for an e-commerce ecosystem’s success (Cennamo, 2021), as we reflect in MR6 on the curation of the ecosystem and related design principles. Decisions on boundary resources are structural for the platform, affect its core architecture, constrain or propel platform evolution, and are therefore difficult to imitate by competing platforms (Blaschke et al., 2019a; Cennamo, 2018).

This research also has certain *limitations*. We could not differentiate between boundary resources provided by platform ecosystem providers and e-business platform ecosystems, as the boundaries between these two categories are increasingly blurred, with retailers and wholesalers in e-commerce also offering software and cloud services (e.g., Amazon, REWE). Hence, we considered five leading examples from both types for our case studies. For focal platforms integrated into enterprise system portfolios (e.g., SAP, Salesforce), it was difficult to determine the boundary resources specific to e-commerce. We only considered software components directly related to the shopping modules. The ten global and European platforms were selected based on their overall dissemination within e-commerce and total revenues. In line with the literature on design principles (Chandra Kruse et al., 2019; Gregor et al., 2020), we strived to codify prescriptive knowledge from successful cases. The underlying shared phenomenon focuses on the exploitation of standardized platform boundary resources in successful e-commerce ecosystems. In addition, we excluded social commerce platforms from our case study analysis, although it is a field of increasing importance for e-commerce. The specifics of social commerce, compared to B2B and business-to-consumer (B2C) commerce, require adjusted design principles for standardization; for example, trust that is fostered by the focal platform is even more important in customer-to-customer (C2C) transactions. Moreover, we did not explicitly consider different maturity stages when selecting our cases (Muzellec et al., 2015; Parker et al., 2016; Reillier & Reillier, 2017). An ecosystem in the launch stage might require less sophisticated boundary resources, as the number of services provided for participants is often limited (Wulfert et al., 2021). Beyond that, we focused on one research stream (i.e., our interactions on the technical and organizational level) when developing our theoretical lens (Foerderer et al., 2019).

## Conclusion and outlook

Based on the theoretical lens of boundary object theory, we developed 19 theoretically grounded and empirically validated design principles for the standardization of focal platforms in e-commerce ecosystems. The design principles address the standardization of the meta-requirements of tailored boundary resources, the openness of the ecosystem, trust and risk, rules and standards, the development environment, and ecosystem curation. In our argumentation, we stressed the importance of developers for e-commerce ecosystems and their requirement of improved standardization. Our design principles simplify the development of external modules and possibly enable multi-homing for developers due to standardization across ecosystems.

Extending our preliminary research, we will conduct interviews with domain experts and knowledgeable senior managers (e.g., consultants, software developers, and platform owners) following the guidelines of Iivari et al. (2021) to further evaluate our design principles in future research. These interviews can also be used to evaluate the relevance of our 19 design principles for different roles at focal platforms and to verify the appropriateness of our design principles for different stages of platform and ecosystem maturity. Future research might also derive patterns of design principles for types of focal platforms in e-commerce ecosystems (e.g., platform ecosystem providers and e-business platform ecosystems) and specific boundary resource archetypes. It might also be interesting for future research to consider the role of boundary resources in unsuccessful cases. As we explicitly focused on third-party developers when deriving design principles for the standardization of digital business ecosystems, future research might address the requirements of different ecosystem participants and summarize prescriptive knowledge. An interesting avenue for future research would be the aggregation of several sets of design principles for different ecosystem participants for a holistic design theory integrating the perspectives of all relevant participants. In the same vein, multi-dimensional modeling of all participants’ perspectives is necessary to improve information systems’ quality. Applying actor-network theory can lead to a deeper understanding of the relations between participants in an ecosystem. As our research is concerned with B2B and B2C commerce, our design principles can be evolved by future research to cope with the specifics of social commerce. Future research may also employ other theoretical lenses when developing design principles (e.g., a design and engineering perspective, platform governance).

## Appendix

### Appendix 1: Cases of the multiple-case study

A central aspect of our analysis for the development of design principles was the examination of Platform Ecosystem Providers (A) and E-Business Ecosystem Platforms (B). In the following section, we present further information for each of the considered cases. Key figures for type A are summarized in Table 4 and for type B in Table 10. An extract of sources analyzed for the derivation of design requirements is presented in Tables 5, 6, 7, 8, 9 and 11, 12, 13, 14, 15. We also provide a table summarizing the contribution of our selected cases to our 19 design principles in Table 16.

**Platform ecosystem provider (A)**

**Table 4:** Overview of key figures of the considered platform ecosystem provider

	Shopware	Magento	SAP Commerce Cloud	Salesforce Commerce Cloud	OXID eShop
Establishment	2004	2008	2012*	1999	2003**
Number of employees	300	375	---	49,000	51–200**
Total turnover (billion euro)	0.02	84.7	---	14.5	---
Customers	Aston Martin Borussia Dortmund Melitta Thyssenkrupp	Bauhaus Burger King Frankfurt Airport Shopping Seat	ALBA Gruppe Bosch New Era Cap Yell	Adidas Amazon Web Services Barclays Coca-Cola	AIDA Bitburger Hellweg Mercedes-Benz
Service costs for companies	0 € Starter Edition --- From 199 € per month (or one-off 2,495 €) Professional Edition --- From 2,495 € per month (or one-off 39,995 €) Enterprise Edition	0 € Community Edition --- One-off from 15,000€ to more than 100,000 € Enterprise Edition	Price according to offerStandard Edition --- Price according to offer Professional Edition	Various prices per user per month depending on application area and requirements	0 € Community Edition --- One-off 2,900 € Professional Edition --- One-off 18,000 € Enterprise Edition (B2C) --- One-off 32,000 € Enterprise Edition (B2B)

* = Year of publication of the SAP Cloud Platform.

** = Values refers to the OXID eSales AG.

**Salesforce Commerce Cloud** is an e-commerce platform that bridges B2C and B2B commerce to provide a comprehensive, seamless omnichannel solution across mobile, social, web, and stationary retail. The platform pushes a strong cloud focus and integration with the Salesforce ecosystem with its ability to develop and sell custom apps. Customers of the platform ecosystem include OSRAM, ZWILLING, Adidas, and Thomas Sabo.

**Table 5:** Salesforce examples on analyzed sources

Content	URL
Webpage	https://sforce.co/3Fh6ClT
Developer Portal	https://sforce.co/3soFgHd
Administrator Portal	https://sforce.co/3qlXhDs
API documentation	https://sforce.co/3yNGzR5
Access to GitHub Repositories	https://sforce.co/3FhJ7tf
GitHub Salesforce	https://bit.ly/3sqddHg
Commerce Cloud Partner Marketplace	https://sforce.co/30NtJFZ
Salesforce App Exchange	https://sforce.co/3J8gh0r
Roadmap for its own product	https://sforce.co/3yNYGXd
Roadmap Resources and Templates	https://sforce.co/3qi96ds
Workflows and approval processes	https://sforce.co/3FhE3oF
Create a Workflow Rule	https://sforce.co/3H0OxJA
SFRA Architecture	https://sforce.co/3pdSO6j
Prototyping UX Solutions in Salesforce	https://bit.ly/3J3JNVr
Discover and Use Prototyping Tools for Lightning Experience	https://bit.ly/33H0sO9
Rapid Prototyping with the Design System Starter Kit	https://bit.ly/3su5zvH
Salesforce commerce cloud app, portal, and web templates	https://sforce.co/3peGIty
Salesforce kicks off Dreamforce with 6 ‘code-free’ Y platform tools	https://bit.ly/3J5AhRr
Demystifying Salesforce Commerce Cloud Development	https://bit.ly/3JbPZuC
Lightning Web Components	https://sforce.co/3Egoigm
Webpage Mindsquare	https://bit.ly/3pfN5wH
Salesforce guides	https://sforce.co/3yQ9T9I

**Magento** is a leading provider of e-commerce solutions for enterprises in both B2C and B2B, and was acquired in 2018 by Adobe, which integrated the platform into its omnichannel solutions. The ecosystem includes a global network of solution and technology partners, a worldwide active developer community, and an online marketplace for extensions such as marketing automation or payment provider integration. The platform provides intelligent AI-based commerce capabilities, SOAP and REST support, a fully automated testing framework, and sample code for rapid third-party application development. Magento has been adopted by more than 260,000 companies worldwide, including ASUS, Coca-Cola, HP, and Nestle.

**Table 6:** Magento examples on analyzed sources

Content	URL
Webpage	https://adobe.ly/33OZcZt
Developer Portal	https://bit.ly/3eeKmNH
API-Documentation	https://bit.ly/3eeKmNH
Magento Marketplace Themes	https://bit.ly/3pg2n4L
Themes at external developers	https://bit.ly/3Fk923h
External Libraries	https://bit.ly/3pcr81E
Roadmap	https://bit.ly/3EfYJfw
Status overview of Magento Modules	https://bit.ly/3H0PykS
Semantic Versioning 2.0.0	https://bit.ly/3pi1h8I
Adobe Commerce Interactive Product Experience	https://adobe.ly/32q7sys
External Demo-Store	https://bit.ly/3siXEkG
Recommendations SDK	https://bit.ly/3Fj4RF4
Using Grunt	https://bit.ly/3Ef3EgI
Locate templates, layouts, and styles	https://bit.ly/3ee4xLS
Web API functional testing	https://bit.ly/3Eiohsm
Getting Started with our Web APIs	https://bit.ly/3qiaE7g
GraphQL Overview	https://bit.ly/3JaaojC
Marketplace EQP API	https://bit.ly/3yN7QD1
Create Magento Account	https://bit.ly/3skPqbP
Adobe Commerce 2.4 Developer Guide	https://bit.ly/3eeKmNH
B2B Developer Guide	https://bit.ly/3H66SF7
Key words for use in RFCs to Indicate Requirement Levels	https://bit.ly/3Eiai5V
Fundamental coding and application design principles	https://bit.ly/3qeiLSD
CQRS	https://bit.ly/32o0h9K
Overview of UI components	https://bit.ly/3mpgBhL

**SAP Commerce Cloud** is an omnichannel platform for B2B and B2C commerce with a strong focus on standardizing key e-commerce functions such as product sales and promotion. The platform solution offers a standard variant and an extended omnichannel solution that can be hosted on-premises or in the cloud. Companies using Commerce Cloud as an e-commerce solution include Office DEPOT, The Shilla Duty Free, Maui Jim, and New Era Cap.

**Table 7:** SAP Commerce Cloud examples on analyzed sources

Content	URL
Webpage	https://bit.ly/3pglcVf
Build and Deploy Your First SAP Commerce Cloud Project	https://bit.ly/3H1otOf
Overview and Key Benefits of SAP Commerce Cloud (1/2)	https://bit.ly/32lrM3S
Overview and Key Benefits of SAP Commerce Cloud (2/2)	https://bit.ly/3EhI2A0
Builds and Cloud Environment Setup	https://bit.ly/3mo29q9
Commerce Webservices	https://bit.ly/3yOI15S
Business and developer area	https://bit.ly/3yOI15S
Micro learning videos	https://bit.ly/3mmStfO
Learning Journeys	https://bit.ly/3J893Kc
SAP Community for Commerce Cloud	https://bit.ly/3peFr5I
SAP Help Portal	https://bit.ly/3H2Pf9e
Building the future of DTC e-commerce, one hack at a time	https://bit.ly/3H1oHVB

**Shopware** is an e-commerce platform with a primary focus on the online store component and integrated content management system. The system is available both in a commercial version and as an open-source variant. Technologically, the system follows the API-first approach in both versions and thus decouples front-end and back-end through a headless commerce architecture approach.

**Table 8:** Shopware examples on analyzed sources

Content	URL
Webpage	https://bit.ly/3phQ6ge
GitHub Shopware	https://bit.ly/3FoHAle
Developer Portal	https://bit.ly/3qdOxPA
Roadmap	https://bit.ly/3st9Ter
Coding Standards	https://bit.ly/3yKU046
Debugging	https://bit.ly/3ebi86q
Rest API – Basics	https://bit.ly/3yO3g7R
Integrations/API	https://bit.ly/32mHig2
API first – Core architecture	https://bit.ly/3eediW8
GitHub – DEV	https://bit.ly/3slGin5
GitHub – Production Template	https://bit.ly/32r3kOJ
GitHub – Overall	https://bit.ly/3su8hkR
COVID-19 Hackathon	https://bit.ly/3mmZxJr

**OXID eShop** is an online store system located in Germany and is available in both commercial and open-source versions. The system currently has more than 2,100 active customers, mostly from Germany and the European region, and can be related to the standard implementation of German and European law. In addition to legal security, the store provider relies on the provision of certified plugins in the context of building trust within the ecosystem**.**

**Table 9:** OXID eShop examples on analyzed sources

Content	URL
Webpage	https://bit.ly/3J6KT2w
OXID e-Shop test	https://bit.ly/3pflZpo
Developer Portal	https://bit.ly/3Elcfyi
Software Repository	https://bit.ly/3EidcHR
Release Plan	https://bit.ly/3yNK6yJ
Module Installation and Activation	https://bit.ly/3J3NOsZ
SDK	https://bit.ly/3yKUUO2
Exchange possibilities	https://bit.ly/3EgMm2w
OXID AI	https://bit.ly/3eaOFK7
Customizing	https://bit.ly/3eaOFK7
Developer Registration	https://bit.ly/3Fdnbzg
Support	https://bit.ly/3H2Ridq
Partner	https://bit.ly/3mmUxo4
Academy	https://bit.ly/3H18VtV
Interview with Gültekin Koc	https://bit.ly/3z0hJ0B
Whitepaper	https://bit.ly/3pfxAoz
Coding Days	https://bit.ly/3eaJ1Yv
Best Practice	https://bit.ly/3ee7Lis

## E-Business platform ecosystems (B)

**Table 10:** Overview of key figures of the considered e-business platform ecosystems

	Amazon	Walmart	Ebay	Etsy	Otto
Establishment	1994	1962	1995	2005	1949
Number of employees	1.298.000	2.200.000	13.300	1.240	49.895
Total turnover (billion euro)	326,9	473,5	9,1	1,4	15,6
Turnover from E-Commerce (billion euro)	167,1	26,2	s. a	s. a	10
Likes on Facebook (million) (as of June 2021)	29	33	11	4,1	1,2
Traffic* (million page views per month) (as of June 2021)	2700	474.5	891,5	381	57,39**

* = Refers to the website with the extension “.com”

** = Refers to "otto.de"

**Amazon** is the global market leader in online retail. In addition to its own trading, Amazon also offers seller accounts via the Marketplace, which are aimed at the B2B and B2C markets. In B2C, Amazon has 300 million customers worldwide. Moreover, more than 1/3 of the top 500 online retailers in Germany additionally sell their products on Amazon Marketplace. The turnover of the Marketplace has already been higher than the turnover of the proprietary trade in Germany since 2016.

**Table 11:** Amazon examples on analyzed sources

Content	URL
Webpage	https://amzn.to/3skSdSl
Developer Portal	https://amzn.to/3H2Z9ra
API Documentation	https://bit.ly/3Jk7kBF
WMS for Integrators Registration Process	https://bit.ly/32hdXnt
FAQ	https://amzn.to/3FjCbLY
Developer Forum	https://amzn.to/3eeOA81
Fist Steps with MWS	https://bit.ly/3J0PYt8
FAQs	https://bit.ly/3miw0jZ
Amazon Business Developer Guide	https://bit.ly/3J7RrOr
Client-Library in PHP	https://bit.ly/3et2y6F

**Walmart** is the world's largest retailer in terms of sales ($559.2 billion). Since 2000, the corporation has pursued a stringent e-commerce strategy (walmart.com). By the acquisition and integration of different marketplaces during the past five years, the company has built a direct competition to Amazon. The Walmart Marketplace enables third-party vendors to use Walmart's coverage as well as its logistics infrastructure.

**Table 12:** Walmart examples on analyzed sources

Content	URL
Webpage	https://bit.ly/3phT6cu
Developer Portal	https://bit.ly/3mncbrC
API documentation	https://bit.ly/3q9wgCW
Walmart I/O	https://bit.ly/3ElBdNT
API PHP Client	https://bit.ly/3EhfCpV
SDK	https://bit.ly/3FkX0GQ
New Marketplace Item Spec v4.2	https://bit.ly/3pcvPIO
Item setup workflow	https://bit.ly/3mpxf0S
API Sandbox	https://bit.ly/3mndaYQ
Onboarding	https://bit.ly/3Fgvqe4
Affiliate Marketing API	https://bit.ly/3yNdkh5
Support via stackoverflow	https://bit.ly/3H1bjkn
Walmart Help	https://bit.ly/3H2FZBP
Walmart Hackathon (US)	https://bit.ly/3EgNuU1
Walmart CodeHers	https://bit.ly/3J1XaW5

**Ebay** is one of the largest e-commerce marketplaces with more than 3.2 billion items sold. The basic idea of the marketplace was the digital adaptation garage sales. Today, the marketplace is primarily characterized by B2C offerings, in addition to the original C2C orientation. Integrating third-party service providers such as payment and shipping service providers (e.g., Paypal, DHL), creates within the company a holistic ecosystem that accompanies the entire selling and buying process.

**Table 13:** Ebay examples on analyzed sources

Content	URL
Webpage	https://bit.ly/3peVr7H
Developer Portal	https://ebay.to/3yQw8we
API-Documentation	https://ebay.to/33Np4F8
GitHub: Ebay	https://bit.ly/3qi6JYe
SDK	https://ebay.to/3pi8KVg
Registrierung Ebay Developer	https://ebay.to/32gNiqH
Techblog	https://bit.ly/3H2RpFH
FAQs und Kontaktkanäle	https://ebay.to/3H1E54j
News	https://ebay.to/3mn5Igt
Feed Beta API	https://ebay.to/33NRVJr

**Etsy** is a global marketplace for handmade or vintage items and craft supplies. The platform was founded in 2005 and, with 4.7 million active sellers, offers over 90 million items for sale (2020). The platform aims to push social interaction of creative buyers with sellers who create or curate items, many of which can be personalized or customized. In this way, Etsy specializes in selling goods purchased in conjunction with special occasions (e.g., wedding, birthday).

**Table 14:** Etsy examples on analyzed sources

Content	URL
Webpage	https://etsy.me/3pp5Eil
Developer Portal	https://etsy.me/3eb2yHZ
API documentation	https://etsy.me/3pcLPdQ
Etsy Ruby Gem	https://bit.ly/3FdE7Wm
Webservice:Etsy	https://bit.ly/3Fkshtx
Etsy Client for REBOL	http://reb4.me/r/etsy
Roadmap v3 API	https://etsy.me/3J7A4x4
jQuery Search App	https://etsy.me/3Eh2cKJ
PuSH Subscription	https://etsy.me/32nSF7g
PuSH Subscriber	https://etsy.me/3stpScr
API: Shop	https://etsy.me/3J8BtUg
API: Treasury	https://etsy.me/3eetWVK
API: Receipt	https://etsy.me/32gRkzl
API: Billing	https://etsy.me/3H1t5nB
API: Listing	https://etsy.me/3EiwY5Z
API: Get Full Access	https://etsy.me/3Fuahgg
Richtlinie für API-Tests	https://etsy.me/3qg5BV5
API: RSS Feeds	https://etsy.me/32kM8ui
Account creation	https://etsy.me/3FjuHZA
API: OAuth Authentification	https://etsy.me/32kMelE
API: Making Requests	https://etsy.me/3mpAaGJ
API: Merchandising	https://etsy.me/3FxwoTr

**Otto** is a German trading and service company with a focus on distance selling. The origins of the company lie in the catalog-based mail order business, and, in the course of digitization, has developed into a platform with more than 5.2 million products, 1500 market partners, and more than 7000 brands. Otto handles the entire purchasing process, including payment processing and product shipping, for its partners, and offers a one-face-to-the-customer approach.

**Table 15:** Otto examples on analyzed sources

Content	URL
Webpage	https://otto.me/3J8Ei7B
Developer Portal	https://otto.me/3mJrICz
API-Documentation	https://bit.ly/3slsPvG
GitHub: Otto	https://bit.ly/3EkKo18
Techblog	https://otto.me/3yQCvzE
Changelog for APIs	https://bit.ly/3slsPvG
About Otto Markt	https://bit.ly/3ejqBVb
Account creation	https://bit.ly/3qixmfw
Otto Markt API	https://bit.ly/3slsPvG
Hackathon Otto IT	https://otto.me/3qiP9n5

**Contribution of case companies to design principles**

In the following we provide an overview of the contribution of the case companies selected to our 19 design principles (Table 16).

**Table 16:** Case companies and design principles matrix

	Platform Ecosystem Provider (A)					E-Business Platform Ecosystems (B)				
	Shopware	Magento	SAP Commerce Cloud	Salesforce Commerce Cloud	OXID eShop	Amazon	Walmart	Ebay	Etsy	Otto
DP1	X	X	X	X	X	X	X	X	X	X
DP2	X	X	X	X	X	X	X	X	X	X
DP3	X	X	X	X		X				
DP4	X	X	X		X	X	X	X	X	X
DP5	X			X				X	X	
DP6		X		X						
DP7		X	X	X						
DP8	X	X		X		X				
DP9	X	X		X	X	X				X
DP10	X	X		X	X			X		X
DP11		X		X	X		X	X		
DP12			X	X			X			
DP13		X	X	X		X	X			X
DP14	X	X		X	X		X		X	
DP15		X	X	X						
DP16		X				X			X	X
DP17		X	X	X	X	X	X	X	X	X
DP18	X	X	X	X						
DP19	X	X		X	X				X	

X = Contribution to a Design Principle

### Appendix 2: Derivation of the theoretical lens

To conduct a purposeful review, we developed a theoretical lens as part of the review of the theoretical basis for analyzing the literature and empirical cases. The foundation for the developments of the lens were the works of Ghazawneh (2012) and Dal Bianco et al. (2014). Development of the lens followed an iterative, collaborative three-step process (Fig. 6). Within the first iteration step, boundary resources were extracted from the literature and irrelevant concepts were extracted. Building on this, in the second step the overlapping concepts were aggregated, and duplicates excluded. Finally, in the third step, specialized terms such as "debugging tools" were generalized to cover as broad a field of literature as possible.

## Footnotes

1 With direct and indirect network effects, the value of an e-commerce ecosystem for ecosystem participants increases with the number of similar participants (direct network effects) and the overall size of the network (indirect network effects). This is due to the higher potential of exchange (e.g., products, product reviews, knowledge) between these participants (Eisenmann et al., 2006; McIntyre & Srinivasan, 2017; Rochet & Tirole, 2003). A prerequisite for indirect network effects is the presence of bidirectional cross-group-side network effects (Hagiu & Wright, 2015; Shapiro & Varian, 1998).

2 A single ecosystem participant may also take on several roles within a single ecosystem.
